# Heterogeneity between and within Strains of *Lactobacillus brevis* Exposed to Beer Compounds

**DOI:** 10.3389/fmicb.2017.00239

**Published:** 2017-02-14

**Authors:** Yu Zhao, Susanne Knøchel, Henrik Siegumfeldt

**Affiliations:** Microbiology and Fermentation, Department of Food Science, Faculty of Science, University of CopenhagenFrederiksberg, Denmark

**Keywords:** *Lactobacillus brevis*, hop compounds, manganese, ethanol, fluorescence microscopy, flow cytometry, heterogeneity

## Abstract

This study attempted to investigate the physiological response of six *Lactobacillus brevis* strains to hop stress, with and without the addition of Mn^2+^ or ethanol. Based on the use of different fluorescent probes, cell viability and intracellular pH (pHi) were assessed by fluorescence microscopy combined with flow cytometry, at the single cell level. The combined approach was faster than the traditional colony based method, but also provided additional information about population heterogeneity with regard to membrane damage and cell size reduction, when exposed to hop compounds. Different physiological subpopulations were detected under hop stress in both hop tolerant and sensitive strains. A large proportion of cells were killed in all the tested strains, but a small subpopulation from the hop tolerant strains eventually recovered as revealed by pHi measurements. Furthermore, a short term protection against hop compounds was obtained for both hop tolerant and sensitive strains, by addition of high concentration of Mn^2+^. Addition of ethanol in combination with hop compounds caused an additional short term increase in damaged subpopulation, but the subsequent growth suggested that the presence of ethanol provides a slight cross resistance toward hop compounds.

## Introduction

Beer represents a hostile environment for most microorganisms due to the presence of hop compounds, low pH, ethanol and carbon dioxide as well as the limited amount of oxygen and nutrients (Sakamoto and Konings, 2003; Suzuki, 2011). Nevertheless, some bacteria are still able to spoil beer. *Lactobacillus brevis* is the predominant spoilage organism, and has therefore been studied extensively (Yansanjav et al., 2004; Behr et al., 2007; Suzuki, 2011; Bergsveinson et al., 2015; Schurr et al., 2015a).

Hop compounds are an essential part of beer brewing. Besides imparting a bitter flavor, hop compounds (mainly iso-α-acids) are antimicrobial, and tolerance toward hop is therefore a prerequisite for beer spoilage bacteria (Suzuki, 2011). There are two antibacterial modes of hop compounds. They can act as mobile-carrier ionophores, which cause a decrease in intracellular pH (Simpson, 1993). The minimum inhibitory concentration (MIC) of *trans*-iso-α-acids decrease with pH and increase with concentration of divalent cations, particularly Mn^2+^ (Simpson, 1993). An additional mode of action with hop that influence on transmembrane redox reactions in the presence of Mn^2+^ gradients, was recently demonstrated (Behr and Vogel, 2010). Interestingly,

Haakensen et al. (2009) investigated a wide selection of *Lactobacilli* and *Pediococcus* isolates and found that the addition of ethanol to hop agar plates caused a decreased growth of some isolates, while ethanol protected other isolates against hop compounds.

The traditional method of studying susceptibility of microorganisms, e.g., *Lactobacillus* and *Pediococcus* species toward hop compounds has been used to assess the growth of an isogenic population (Simpson, 1993; Fernandez and Simpson, 1995; Haakensen et al., 2009). However, these culture-based method require extended incubations and some strains even exhibit viable but non-culturable characteristics in traditional laboratory media like the de Man Rogosa Sharpe (MRS) medium (Suzuki et al., 2008). In addition, recent studies showed that even cells from an isogenic population exhibit heterogeneous gene expression and differ in physiological parameters such as growth rate and resistance to stress (Strovas et al., 2007; Ryssel et al., 2013; Zhao et al., 2014). Several studies have also shown that under stress conditions, a minor robust subpopulation can subsequently dominate the overall population (Balaban et al., 2004; Lidstrom and Konopka, 2010; Ambriz-Avina et al., 2014). Therefore, single cell analysis can provide additional insights into microbial behavior under stress from hop compounds.

Fluorescence microscopy (FM) and/or flow cytometry (FCM) have been successfully used for determination of cell population heterogeneity, including live, dead, or intermediate subpopulations (Guldfeldt and Arneborg, 1998; Barbesti et al., 2000; Ambriz-Avina et al., 2014; Stiefel et al., 2015). These observations include morphological changes like cell size and clumping, as well as physiological changes in membrane permeability, intracellular pH and membrane potential at a single cell level (Davey and Kell, 1996; Garcia-Betancur et al., 2012). Morphological information is readily available from microscopy, while a very large number of individual cells can be analyzed by FCM. Hence, the combined methods of FM and FCM can deliver a combination of qualitative and quantitative information that will provide a comprehensive description of subpopulations and even individual cells.

To the best of our knowledge, the response of *L. brevis* toward hop compounds is still not well described at the single cell level. Therefore, the aim of this study was to analyze how individual cells from six different isogenic strains respond to hop compounds by FM and FCM. Furthermore, an interaction with Mn^2+^ or ethanol under hop stress was investigated, in order to further elucidate mechanisms involved in hop tolerance.

## Materials and Methods

### Media and Chemicals

Two different MRS media were used throughout the study. Normal MRS medium (MRS_5.6_, Merck), which was prepared according to the instructions from the supplier. An acidified MRS medium (MRS_4.3_), where pH was lowered to 4.3 using hydrochloric acid. The final concentration of hop compounds (iso-α-acids) in stress experiments was 55.2 μM unless otherwise indicated, which was obtained by addition of a stock solution of 30% (w/w) iso-α-acids in an aqueous solution of potassium (Hopsteiner, New York, NY, USA). Sodium acetate buffer (50.0 mM, pH 4.3, with 0.1 M glucose) was used for experiments where growth was not wanted. A final concentration of either 2.9 μM manganese (corresponding to levels in pilsner lager beer) (Behr and Vogel, 2009) or 265.0 μM manganese (as in MRS), was obtained by addition of a 26.5 mM MnSO_4_ stock solution to sodium acetate buffer.

### Bacteria Strains and Culture Condition

A total of six *L. brevis* strains isolated from beer were included in the present study (**Table 1**). A preculture was started by inoculating 10 mL MRS_5.6_ from a frozen stock culture, and incubated at 30°C overnight. The preculture was subcultured into fresh MRS_5.6_ (inoculation concentration 1%) at 30°C. After 16 h, optical density (OD_600_) of each strain was adjusted with fresh MRS_5.6_ to 1.0, corresponding to approximately 5 × 10^8^ CFU/mL.

**Table 1:** Organisms used in this study.

Abbreviation	Organisms	Source
JK09	*Lactobacillus brevis* JK09	Danish craft beer
JK09--^a^	*Lactobacillus brevis* JK09--	Plasmid cured JK09
HF01	*Lactobacillus brevis* HF01	Danish craft beer
A	*Lactobacillus brevis* A	Danish craft beer
G430^b^	*Lactobacillus brevis* G430	Czech beer
Q	*Lactobacillus brevis* Q	Danish craft beer

^a^*Plasmid cured variant JK09-- was derived by subculturing JK09 in MRS_*5.6*_ containing the plasmid curing agent novobiocin. The plasmid curing was confirmed by the absence of selected plasmid-borne hop tolerant related genes by PCR (results not shown).*

^b^*L. brevis G430 was kindly provided by Dr. J. J. Leisner (Department of Veterinary Disease Biology, University of Copenhagen)*.

### Determination of Growth Activity of *L. brevis*

Hundred microliter standardized culture was inoculated into 10 mL of the following media MRS_5.6_ (control), MRS_5.6_ with hop compounds (MRS_5.6+H_), MRS_4.3_ and MRS_4.3_ with hop compounds (MRS_4.3+H_), separately. The growth curves were assessed in 96-wells microplates by measuring OD_600_ for every 12 h using a Varioskan^TM^ Flash (Thermo Fisher Scientific Oy, Finland) until 168 h. Each well of a microplate was added 200 μL suspension. Microplates were sealed with parafilm (Sigma–Aldrich) to minimize loss of volume and incubated at 30°C until measurement. For each strain, the average OD_600_ value was calculated from 16 wells.

### Fluorescent Staining

To investigate viability in individual cells, cells were stained with SYTO 13 (Molecular Probes, Thermo Fisher scientific) and Propidium Iodide (PI, Molecular Probes, Thermo Fisher scientific). 1 mL of an overnight culture was incubated with SYTO 13 (final concentration 10.0 μM) and PI (final concentration 15.0 μM) simultaneously at 30°C for 30 min in the dark. The suspensions were subsequently diluted 20 times to an approximate concentration of 10^7^ cells/mL with saline solution (NaCl, 0.9%, w/w). All dual stained cell suspensions were kept on ice until further analysis.

For pHi measurement, cells were stained with 5(6)-Carboxy-2, 7-dichlorofluorescein diacetate succinimidyl ester (CDCFDA-SE, 3.9 mM, Molecular Probes, Thermo Fisher scientific). The CDCFDA-SE staining procedure used in this study was modified from Shabala et al. (2006). Briefly, 1mL standardized culture was harvested by centrifugation (10,000 × *g*, 5 min), and the pellet was re-suspended in 980 μL phosphate buffered saline (PBS, 50.0 mM, pH 7.4) with 10 μL 1.0 M glucose and 10 μL CDCFDA-SE. The cell suspension was incubated at 30°C in the dark for 30 min.

### Sample Preparation for Viability Assessments and Cell Size Comparisons

A standardized culture (200 μL) was inoculated in 20 mL MRS_4.3_ and MRS_4.3+H_, separately, and incubated at 30°C. A 1 mL sample was taken every 6 h until 48 h and then every 24 h until 96 h. Cells were centrifuged at 10,000 × *g* for 5 min and the pellets were re-suspended in 0.5 mL saline solution and stained with SYTO13/PI as described above. Subsequently, samples were analyzed by FM and/or FCM. For FCM, a positive control (fresh overnight culture, live cells) was stained with SYTO 13 and a negative control (dead cells) was stained with PI. For the negative control, preliminary results using 70% ethanol to kill all the cells, but this method caused cell aggregation in FCM analysis. Therefore, a combination of 552 μM hop compounds and 20% ethanol (v/v) was used to obtain a fully permeabilized population of single cells, where almost 100% cells were stained with PI, which was confirmed by FM.

### Sample Preparation for pHi Measurement

Cells from overnight culture were stained with CDCFDA-SE, centrifuged at 10,000 × *g* for 5 min and the pellet was re-suspended in 1mL sodium acetate buffer. Subsequently, the suspension was divided into two tubes, centrifuged at 10,000 × *g* for 5 min and re-suspended into 500 μL sodium acetate buffer with and without hop compounds. Images were acquired after 1, 12, and 24 h by FM. Samples were kept at 30°C in the dark until image acquisition.

**FIGURE 1:**
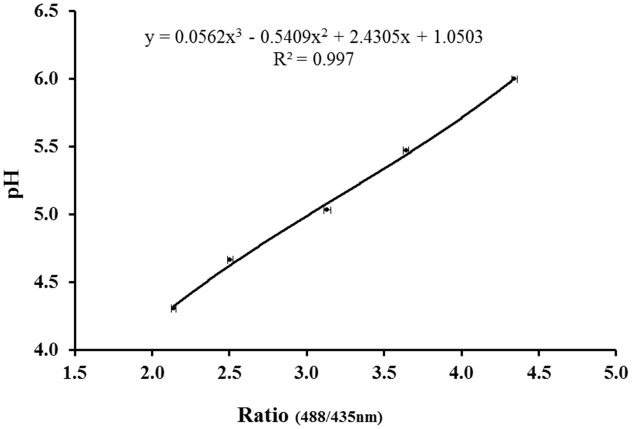
**Calibration curves for pHi determination of six *Lactobacillus brevis* strains**. The values are average of at least 100 cells of all six strains with error bars (SEM). A third degree polynomial was fitted to the calibration points.

To construct a calibration curve for pHi, stained cells were exposed to 70% ethanol for 30 min to achieve fully permeabilized cells. Aliquots of permeabilized cells were then re-suspended in sodium acetate buffer with pH 4.3, 4.7, 5.0, 5.5, and 6.0. Since all six strains exhibited similar ratio values at a given pH, it was decided to construct one standard curve. The average ratio of at least 100 cells for each pH value was plotted against the Ratio _(488/435__nm)_, and the standard error of the mean (SEM) was calculated (**Figure 1**).

### Manganese Addition

1.5-mL standardized culture was centrifuged at 10,000 × *g* for 5 min and re-suspended in 1.5 mL of acetate buffer. For viability assessment, the suspension was divided into three tubes, followed by centrifugation (10,000 × *g*, 5 min), and the pellets were re-suspended in acetate buffer with hop compounds, acetate buffer with hop and 2.9 μM manganese, as well as acetate buffer with hop and 265.0 μM manganese, respectively. Cell suspensions were incubated at 30°C for 2.5 h, stained with SYTO 13/PI for 0.5 h, and analyzed by FCM. For pHi measurement, the suspension was first stained with CDCFDA-SE, then divided into three tubes, centrifuged at 10,000 × *g* for 5 min and the pellets were re-suspended in the three solutions described above. Cell suspensions were incubated at 30°C for 3 h before being analyzed by FM.

### Ethanol Addition

OD_600_ of all strains were measured in MRS_4.3_ and MRS_4.3+H_ with and without addition of ethanol (4.6%, v/v), according to the method described above. For viability assessment, 2 mL standardized culture was centrifuged at 10,000 × *g* for 5 min and the pellet was re-suspended in 2 mL acetate buffer. The suspension was divided into four tubes, followed by centrifugation (10,000 × *g*, 5 min) and the pellets were re-suspended in acetate buffer with and without hop compounds and ethanol. Cell suspensions were incubated at 30°C for 2.5 h, stained with SYTO 13/PI for 0.5 h, and then analyzed by FCM.

### Fluorescence Microscopy and Data Analysis

For viability assessment, a 63× Plan-Apochromat, N.A 1.4 (Zeiss) was used. The green fluorescence (SYTO 13) was recorded by excitation of 470–490 nm and emission of 515–565 nm. The red fluorescence (PI) was recorded by excitation of 515–565 nm and emission of 610–680 nm.

For pHi measurement, the method was the same as previously described (Shabala et al., 2006). Briefly, the two excitation wavelengths were 435 and 488 nm, and emission was collected by a 515–565 nm bandpass filter. Images were acquired by using the Metamorph 7 software (Universal imaging Corp., West Chester, PA, USA) and analyzed with the free image analysis software ImageJ [version 1.48; National Institutes of Health (NIH), Bethesda, MD, USA^1^].

### Flow Cytometry and Data Analysis

Samples were counted and analyzed by a BD FACS Jazz Cell Sorter with a 488 nm argon ion laser. Green fluorescence emitted from SYTO 13 stained cells was collected with bandpass filter 530 ± 20 nm, whereas the red fluorescence emitted form PI stained cells was collected with bandpass filter 692 ± 20 nm. Events were collected by triggering on the side scatter channel. A 1 mL cell suspension was added into a plastic tube and 100.000 events per sample were recorded. Data were collected and stored in BD FACS Software sorter software (BD Biosciences, USA). The collected data were analyzed with the FlowJo_V10 (Tree Star, Inc. USA). For subpopulation assessments, four quadrants were defined clockwise starting from the top-left one as Q1, Q2, Q3, and Q4 on the dot plot images. For cell size comparisons, the histogram of the forward scatter channel (FSC) was used.

### Data Analysis

When growth experiments were performed in several replicates, the average and the standard deviation were calculated. In the FCM experiments, 100.000 cells were analyzed to obtain a large sample, and the proportions of subpopulations were generated by FlowJo_V10 software. In the intracellular pH experiments, the calibration curve from section “Sample Preparation for pHi Measurement” was used to calculate the pHi. Subsequently, the average of at least 20 individual cells was calculated, and the individual cells were manually ascribed to predefined pHi intervals.

## Results

### Growth Behavior of *L. brevis* Strains

**FIGURE 2:**
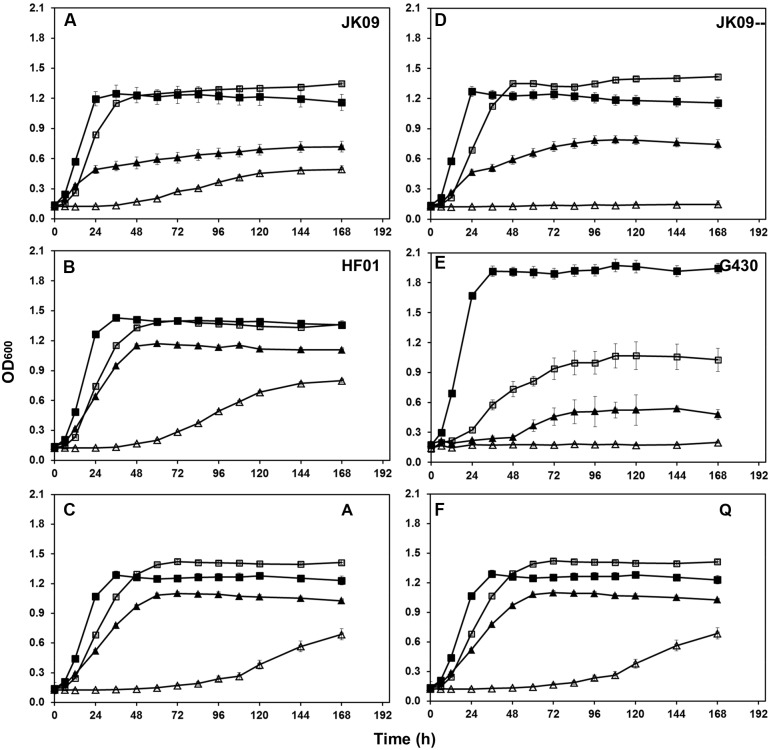
**Growth curves of *L. brevis* exposed to hop compounds in MRS adjusted to different pH values**. Cells were cultured at 30°C in MRS_5.6_ (control,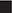), MRS_5.6+H_ (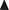), MRS_4.3_ (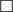), MRS_4.3+H_ (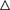). **(A–F)** JK09, HF01, A, JK09--, G430 and Q. The values are the means of OD_600_ of 16 wells in 96-wells microplate, and the error bars indicate the standard deviation.

Growth curves of six strains were obtained under normal conditions, under low pH stress, under hop compounds stress as well as under the combined stress condition (**Figure 2**). All six isolates grew well in normal MRS_5.6_, with G430 reaching the highest OD_600_ (**Figure 2E**). In MRS_4.3_, five strains had a slightly prolonged lag phase, but with the same growth rate and the same final OD_600_. Only G430 was significantly inhibited by the low pH alone (**Figure 2E**). In contrast, all the bacteria exhibited poor growth under hop stress. The combined stress was even more inhibitory. In MRS_4.3+H_, JK09--, G430 and Q were so inhibited that they did not produce any change in optical density during the experiment (168 h), and these strains were consequently designated as hop sensitive strains. JK09, HF01 and A were comparatively tolerant to hop compounds in MRS_4.3+H_, and these strains were designated as hop tolerant strains. However, the lag phase and growth rate of the hop tolerant strains were dramatically affected, particularly for strain A. As the antibacterial effect of hop compounds was mostly pronounced at pH 4.3, a typical pH for beer, pH 4.3 was used for further investigations.

### Viability Assessment of *L. brevis* Cells by FM Analysis

**FIGURE 3:**
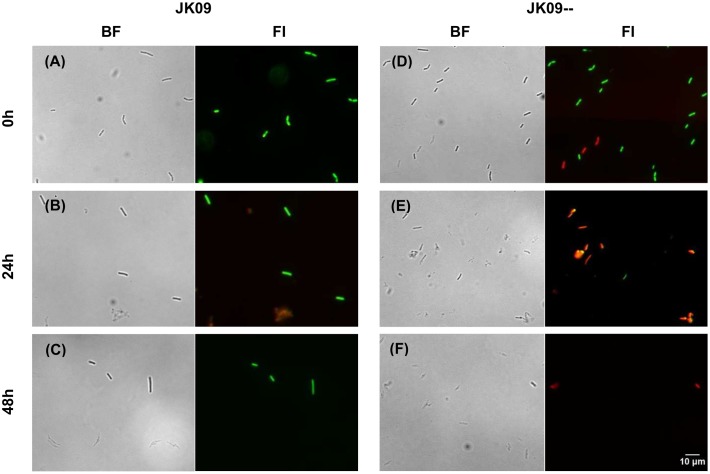
**Cells of JK09 (A–C)** and JK09-- **(D–F)** exposed to hop compounds in MRS_4.3_ after 0, 24, and 48 h, stained with SYTO 13 and PI and analyzed by fluorescence microscopy (FM). BF, bright-field; FI, fluorescent image. For fluorescent images, cells stained with SYTO 13 are labeled green, while cells stained with PI are labeled red.

Viable cells could easily be distinguished from membrane damaged cells by dual-staining with SYTO 13 (green fluorescence) and PI (red fluorescence) using FM. At the beginning of the experiment (*T* = 0 h), most cells of both JK09 and JK09-- showed bright green fluorescence (**Figures 3A,D**). After incubation in MRS_4.3+H_ for 24 h, fuzzy, weak red fluorescent clusters of cells could be observed for both strains, and some cells even appeared unstained (**Figures 3B,E**). But most fluorescent cells of JK09 were labeled green (**Figure 2B**) while most cells of JK09-- were red (**Figure 3E**). After 48 h, the same pattern could be observed with the exception that most of the clusters were not stained with any fluorescent dye (**Figures 3C,F**).

### Viability Assessment and Cell Size Comparison of *L. brevis* Cells by FM Analysis

**FIGURE 4:**
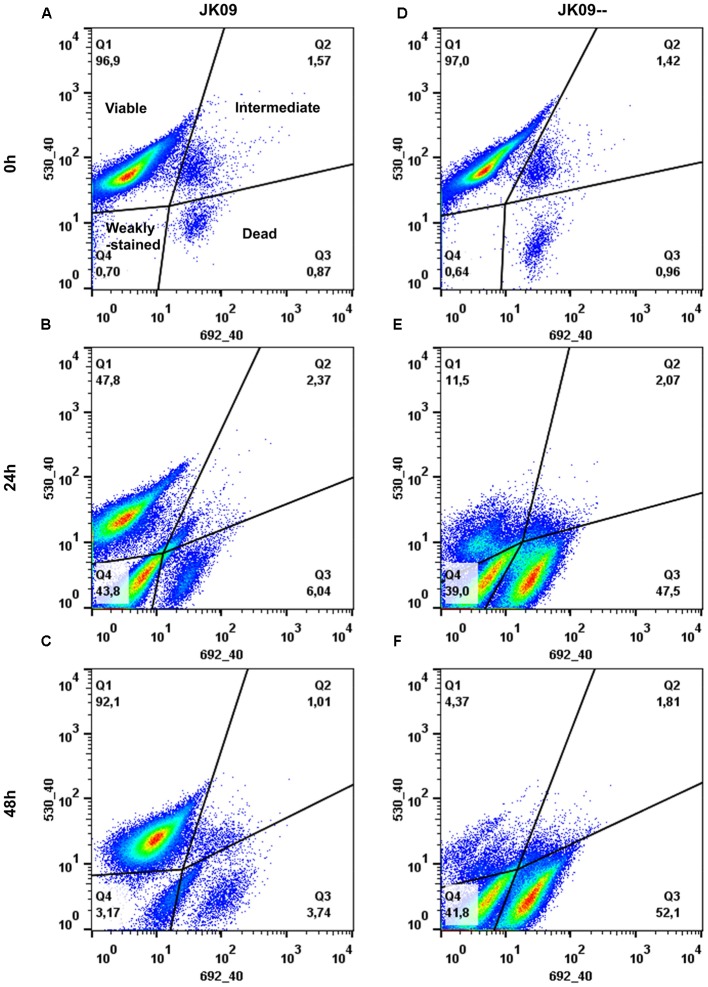
**Density plot images of JK09 (A–C)** and JK09-- **(D–F)** exposed to hop compounds in MRS_4.3_ after 0, 24, and 48 h, analyzed by flow cytometry (FCM). The SYTO 13 signal was recorded in the green channel (530_40), the PI signal was recorded in the red channel (692_40). Events in different quadrants corresponds to different populations: viable cells (Q1), intermediate population with damaged membranes (Q2), dead cells (Q3) and weakly stained cells (Q4), the number in each quadrants represents the relative percentage of each population.

In order to investigate the viability of *L. brevis* under hop compounds stress at a single cell level, stained cells were additionally analyzed with flow cytometer. Four different quadrants were recognized, which represents cells in different physiological states. Quadrant 1 (Q1) is the subpopulation of viable cells with intact membranes that only stained with SYTO 13, Q2 represents an intermediate subpopulation which corresponds to viable cells but with damaged membranes, Q3 describes the subpopulation of dead cells, which stained strongly with PI, Q4 consists of weakly stained cells after hop treatment (**Figure 4A**).

**FIGURE 5:**
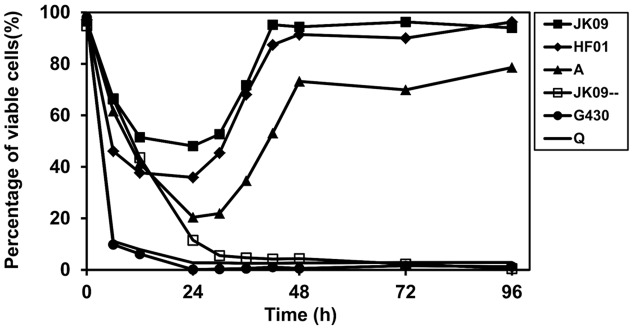
**Change in proportion of the viable subpopulation of *L. brevis* exposed to hop compounds in MRS_4.3_ over time by flow cytometric analysis**. The percentage of viable cells corresponds to the number in Q1 in the density plot images.

The proportion of viable cells for all the strains without hop compounds treatment were above 90% until 72 h followed by a small reduction after 96 h (results not shown). The hop compounds caused an obvious reduction in viable cells in the first 24 h in all strains, especially for the sensitive strains (**Figures 4** and **5**). The viable proportions of JK09, HF01, and A dropped to 48, 35, and 20%, respectively, after 24 h exposure (**Figures 4B** and **5**; Supplementary Figure S1), most of the cells were situated in Q3 and/or Q4 instead (**Figure 4B**; Supplementary Figure S1). However, the proportion of viable cells of these three strains increased markedly during the next 24 h, where the viable proportion of JK09 and HF01reached the same level as in the control experiments after 48 h (**Figures 4C** and **5**; Supplementary Figure S1). On the contrary, the proportion of viable cells in the hop sensitive strains JK09--, G430 and Q continued to decrease after 24 h, and there were almost no viable cells detected after 48 h (**Figures 4E,F** and **5**; Supplementary Figure S1). The proportion of viable cells did not change from 48 to 96 h for any of the strains (**Figure 5**).

**FIGURE 6:**
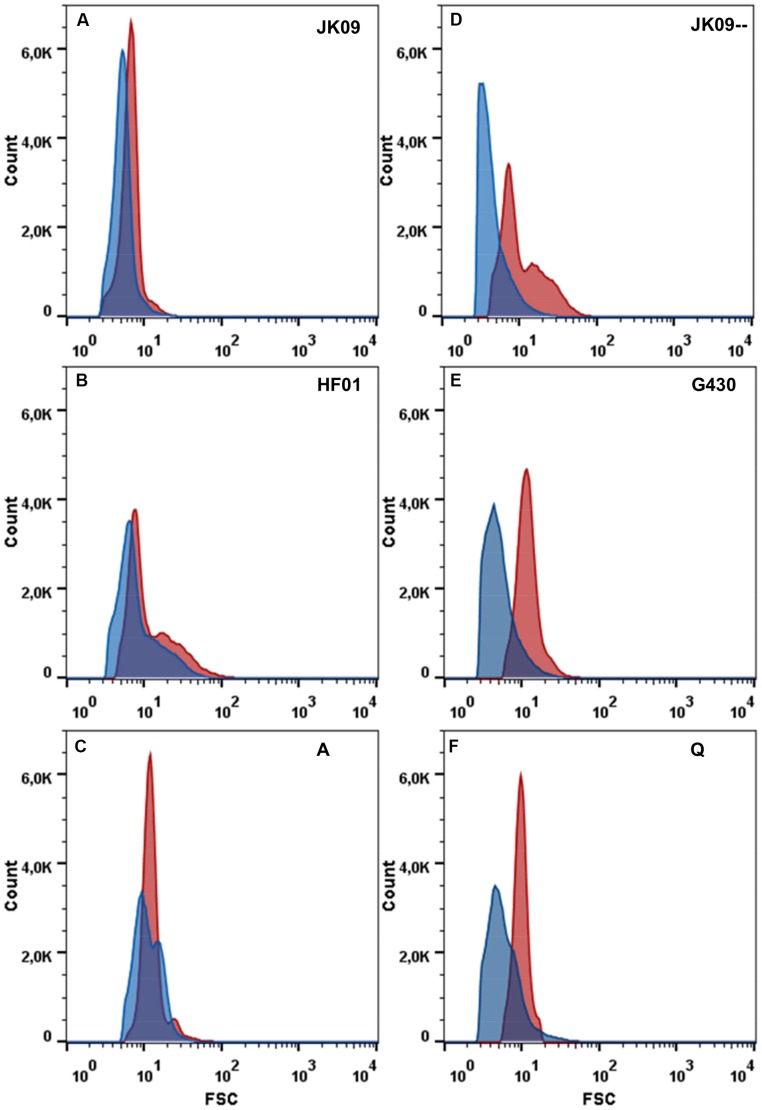
**Cell size distribution of *L. brevis* in MRS_4.3_ after 48 h incubation determined by the forward scatter channel (FSC) from FCM**. **(A–F)** JK09, HF01, A, JK09--, G430 and Q. The red histogram corresponds to cells incubated without hop compounds, while the blue histogram corresponds to cells incubated with hop compounds.

The histogram of the FSC from the FCM analysis reveals the distribution of cell sizes after 48 h (**Figure 6**). Some of the strains revealed a homogenous distribution of cell sizes in the control experiments, e.g., JK09 and Q (**Figures 6A,F**), where other strains exhibited a more heterogeneous distribution, e.g., HF01 and JK09-- (**Figures 6B,D**). The histogram of hop stressed cells shifted toward lower values for all the strains, which indicates that the average cell size was reduced, compared to the control cells, and this tendency was more pronounced for the three sensitive strains.

### Dynamic Change in Intracellular pH of *L. brevis* Cells

Changes in the intracellular pH during 24 h of hop stress in acetate buffer (pH 4.3) were determined by fluorescence microscopy. For clarity, three subpopulations were created according to the pH points of the calibration curve. Cells with 4.3 ≤ pHi < 4.7 have a small or absent ΔpH, which correspond to the population of damaged or dead cells, cells with 4.7 ≤ pHi < 5.0 have a small but noticeable ΔpH, and represent the intermediate population, whereas cells with pHi ≥ 5.0 have a large ΔpH and are regarded as viable cells.

**FIGURE 7:**
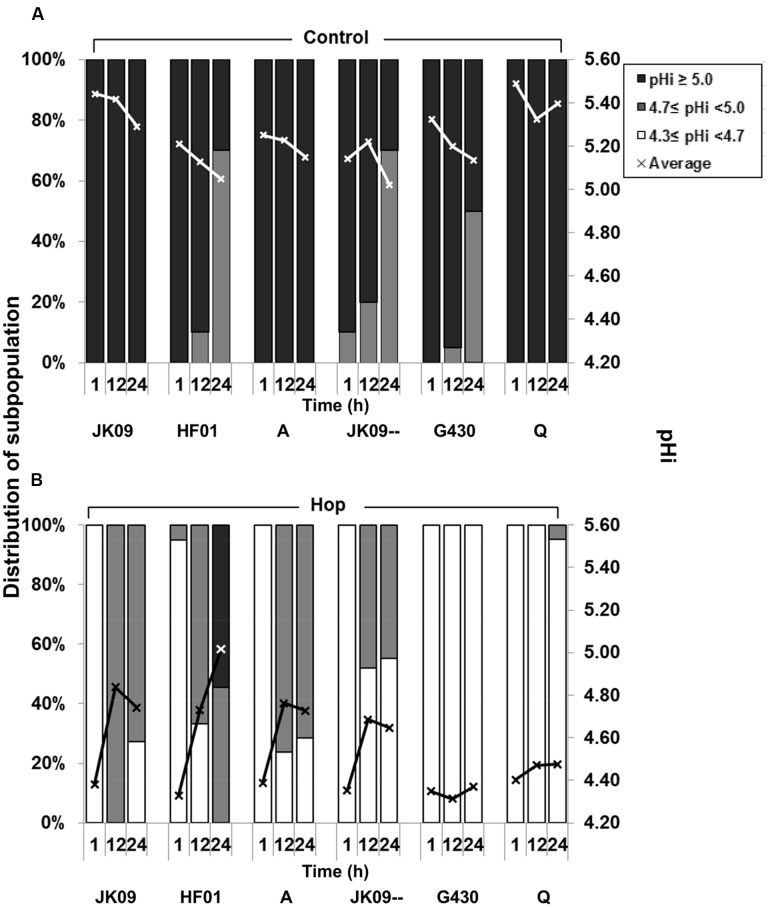
**Distribution of subpopulations of *L. brevis* according to the pHi of individual cells**. In sodium acetate buffer **(A)** and in sodium acetate buffer with hop compounds **(B)** after 1, 12, and 24 h, analyzed by FM. The average pHi value is based on at least 20 individual cells, and the cells were attributed to different subpopulations according to the pH points of the calibration curve.

After 1 h, the average pHi of all six strains in acetate buffer without hop compounds was 5.3 ± 0.1, with all of the individual cells exhibiting a pHi ≥ 5.0 (**Figure 7A**). The only exception was JK09--, which had a minor percentage (10%) in the intermediate population. There was a slight reduction in average pHi for all strains over time, but G430 showed the biggest pHi reduction. Under hop stress for 1 h, a sharp drop in pHi was observed for all six strains, where all cells reached the detection limit of pH 4.3 (**Figure 7B**). During the following 23 h, JK09, HF01, A as well as JK09-- had an obvious increase in pHi. For HF01, the average pHi value after 24 h reached 5.0, and with 55% of the individual cells exhibiting a pHi ≥ 5.0. On the other hand, the average pHi value for Q and G430 was fluctuating around 4.3, with almost all cells in the damaged/dead subpopulation (4.3 ≤ pHi < 4.7) throughout the experiment.

### Protective Effect of Mn^2+^ on *L. brevis* Cells

**FIGURE 8:**
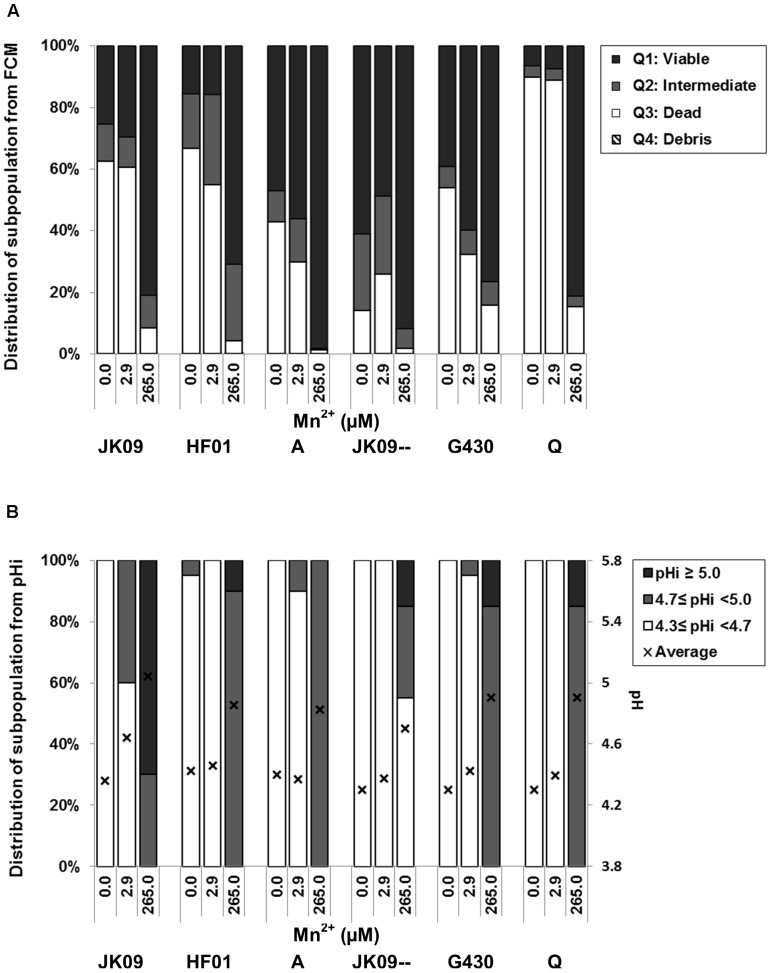
**Distribution of subpopulations of *L. brevis* according to the addition of manganese**. Effects of different concentrations of Mn^2+^ on viability **(A)** and pHi **(B)** of *L.brevis* after exposure to hop compounds in acetate buffer for 3 h. The distribution in **(A)** corresponds to the number in each quadrant of the density plot images. The average of pHi values in **(B)** is based on at least 20 individual cells and the cells were attributed to different subpopulations according to the pH points of the calibration curve.

The impact of manganese on the viability of *L. brevis* was tested in acetate buffer with hop compounds using FCM (**Figure 8A**). After 3 h of exposure to hop compounds, the number of viable cells in acetate buffer without manganese was decreased. There is no clear trend that separated the hop tolerant strains from the hop sensitive strains. After supplementation with the level of Mn^2+^ found in pilsner lager beer (2.9 μM Mn^2+^), there was a tiny increase in the viable and/or intermediate populations of all strains except JK09--. When Mn^2+^ addition was increased to the level found in MRS (265.0 μM), the percentage of viable cells increased significantly in all strains with the largest relative change in strain Q.

As shown in **Figure 8B**, the pHi of all six strains after 3 h exposure was around 4.3, the same value as after 1 h exposure (**Figure 7B**). 2.9 μM Mn^2+^ had limited effect on pHi for most of the strains except JK09, where 40% shifted to the intermediate population. At high level of Mn^2+^ (265.0 μM), a very significant increase in the average pHi was observed in all strains, with 100% individual cells exhibiting a pHi ≥ 4.7, except in JK09--, where 55% individual cells had pHi < 4.7.

### Effect of Ethanol on *L. brevis* Cells

**FIGURE 9:**
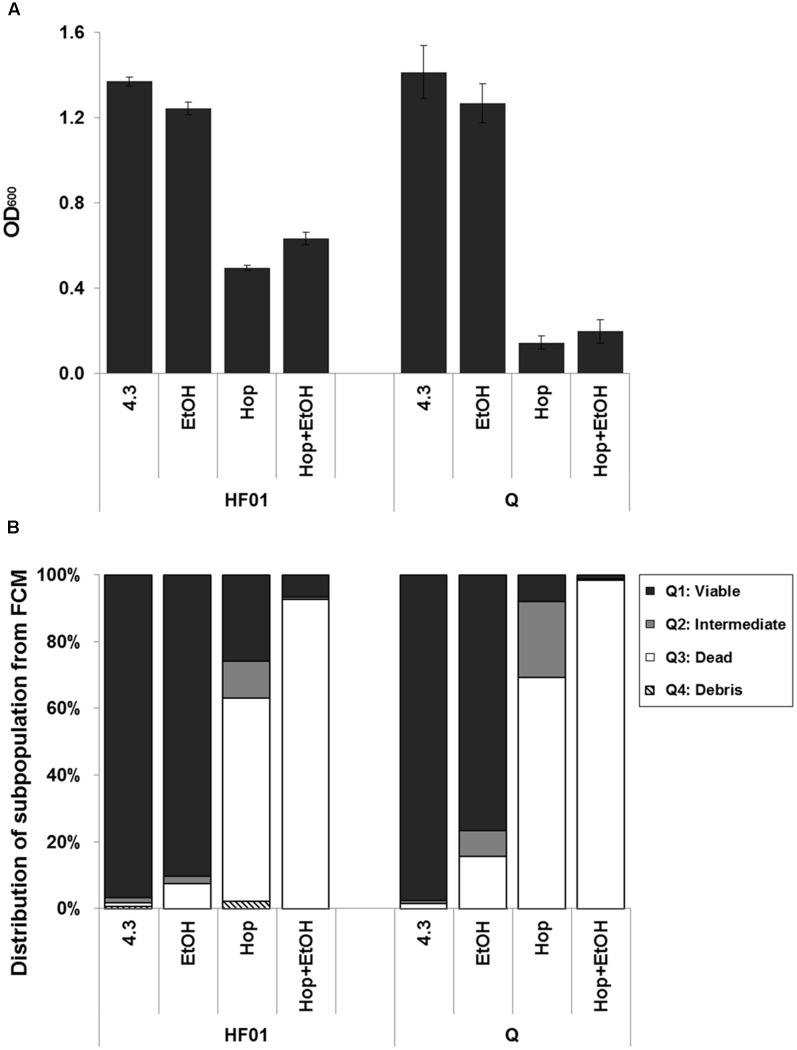
**Effect of ethanol in combination with hop compounds**. The growth experiments of HF01 and Q were performed in MRS 4.3 with and without addition of hop compounds and ethanol, and the OD_600_ values were measured after 96 h incubation **(A)**. The subpopulations of HF01 and Q were analyzed after exposure to acetate buffer with and without addition of hop compounds and ethanol for 3 h **(B)**.

Ethanol (4.6% v/v) inhibited the growth of both hop tolerant strain HF01 and hop sensitive strain Q, as demonstrated by the OD_600_ values after 96 h incubation (**Figure 9A**). This reduction was less pronounced than the effect of hop addition, but the combined addition of hop and ethanol did not show any additional reduction in OD_600_. In fact, the hop tolerant strain grew faster in the combined addition than hop alone, with the largest difference in OD_600_ at 96 h (**Figure 9A**). This phenomenon was also observed in the other two hop tolerant strains (results not shown). The two strains, HF01 and Q, were subsequently analyzed by FCM, and the damaged subpopulations after 3 h showed that the combination of hop and ethanol at this stage was more lethal than hop alone (**Figure 9B**).

## Discussion

Initially, the growth potential of *L. brevis* strains exposed to various combinations of pH, and hop compounds was determined on the population level. Low pH in beer did not by itself inhibit the growth of most of the tested *L. brevis*. However, the low pH had a pronounced influence on the inhibitory effect of hop compounds, and this combination was further explored. The hop tolerant strains JK09, HF01 and A were able to grow slowly during exposure to hop compounds at pH 4.3 (**Figures 2A–C**), but from the growth curves, it is not possible to distinguish whether all cells in the population exhibit a similar response, or whether there are subpopulations that differ in their response to hop compounds.

To answer these questions, we investigated the dynamic physiological response of *L. brevis* cells to hop compounds at a single cell level using FM and FCM. A clear difference in the proportions of subpopulations between hop tolerant and sensitive strains was observed with both techniques. A small but significant subpopulation of the hop tolerant strains exhibited only green fluorescence, while almost 100% of fluorescent cells of the sensitive strains were labeled red after 24 h (**Figures 3**–**5**; Supplementary Figure S1). This indicates that hop compounds causes membrane damage in most cells. Moreover, unstained cells or weakly stained cells could be found after 24 h as well (**Figures 3** and **4**). The reason is still unclear, but ‘ghost cells,’ which has intact cell structures but without nucleic acids, have been reported previously (Booyens and Thantsha, 2014; Léonard et al., 2016). It is conceivable that hop compounds caused such severe damage to the cells that DNA was lost, and consequently the cells were weakly stained.

The hop tolerant strains exhibited a pronounced heterogeneity at the single cell level, which must be a phenotypic variation, as the population is genetically homogeneous. In addition, the growth curves of the tolerant strains exhibited a longer lag phase when exposed to hop compounds (**Figure 2**), which may be partly explained by the reduction of viable cells, and partly by the lower growth rate that was observed. Within the three tolerant strains, the reduction in viable cells was most pronounced in strain A (**Figure 5**), which also had the longest lag phase (**Figure 2C**). This suggests that the reduction in viable cells had a large influence on the observed lag phase. Furthermore, the viable population of the hop tolerant strains increased gradually during the next 24 h (**Figure 5**), which could be explained by the growth of the surviving cells.

Additional morphological information was obtained from the combined analysis. When grown in MRS_4.3+H_ for 48 h, the individual cells retained their rod shape (**Figure 3**), but there was a small reduction in the cell size of hop tolerant strains, compared to grow in MRS_4.3_ (**Figure 6**). This result is in agreement with previous results where *L. brevis* and *L. lindneri* cells appeared as smaller rods when grown in degassed beer compared to growth in MRS (Suzuki, 2011). The hop sensitive strains were much smaller after exposure to hop, probably because they have not divided (**Figure 6**).

Intracellular pH is another parameter to assess stress response (Yansanjav et al., 2004; Smigic et al., 2009; Schurr et al., 2015b). As cell division after staining with CDCFDA-SE will reduce the intensity of cell fluorescence, we decided to test the pHi in sodium acetate buffer, with the same pH, acetate and glucose as MRS_4.3_, but without additional nutrients. This enabled an assessment of recovery with 24 h without cell division. Since most of the tested strains were only slightly affected by the exposure to pH 4.3 (**Figure 2**), the pHi values in pure acetate buffer provided information about the basic pH regulation in an acidic environment. The average pHi of all strains was around 5.3 at *T* = 1 h, with a small decline in the following hours (**Figure 7A**), but this was probably due to a slight acidification of the media (down to pH 4.1) caused by lactic acid production. This is comparable to previous reports that lactic acid bacteria was able to maintain a constant ΔpH of approximately 1.0 (Hutkins and Nannen, 1993). Moreover, G430 had the biggest pHi decrease among the six strains (**Figure 7A**) which corresponds well with the observed acid sensitivity (**Figure 2E**).

Simpson (1993) observed that hop tolerant strains can maintain a higher intracellular pH than sensitive strains. On the other hand, Behr et al. (2006) found that the pHi of a hop-adapted strain also decreased strongly after exposure to hop compounds in acetate buffer for 40 min. In this study, the pHi of all tested strains dropped to pH 4.3 in 1 h (**Figure 7B**), indicating that the initial drop in pHi following exposure to hop compounds is unavoidable. It is a consequence of the protonophoric effects of the hop compounds, but may also be influenced by the reduction in cell size (**Figure 6**), which can increase the intracellular concentration of protons (Vindeløv and Arneborg, 2002). However, we found that the pHi of some individual cells of the hop tolerant strains increased after 12 h (**Figure 7B**). This was also observed in the plasmid cured strain JK09--, but not in the two other sensitive strains. This may be due to the overexpression of membrane-bound ATPase, which pumps protons from the cytoplasm (Sakamoto et al., 2002). Although JK09-- was sensitive to hop compounds in the growth experiments (**Figure 2D**), this strain exhibited a similar recovery in the pHi experiment as the wild type JK09 (**Figure 7B**). This suggests that part of this recovery mechanism can be chromosomal and therefore present in both JK09 and JK09--. This underlines that the mechanism behind hop tolerance is multifactorial, and in this case, partly related to the plasmid borne resistance genes, but also to a certain degree to chromosomal genes. The heterogeneity in the pHi regulation further emphasizes that only a proportion of the cells can withstand the stress from hop compounds.

Previous studies have shown that the MIC of hop compounds increased with an increased addition of Mn^2+^ (Simpson, 1993), and the growth of *L. brevis* could be accelerated by high concentrations of Mn^2+^ (Behr, 2008). We therefore investigated the effect of Mn^2+^ on the viability and intracellular pH by FCM and FM. The viable proportion was slightly increased for most strains by low amounts of Mn^2+^, while it was greatly enhanced for all strains by higher amounts of Mn^2+^ (**Figure 8A**). It has been reported that lipoteichoic acids from the cell wall provides a reservoir of divalent cation such as Mn^2+^. This is very close to the plasma membrane, and therefore the divalent cation probably interact with the negatively charged head groups of phospholipids and decrease the membrane fluidity (Asai et al., 2000; Behr, 2008). It is conceivable that this can reduce the intrusion of hop compounds into cells, and thereby reduce the toxicity of hop compounds. It also has been demonstrated that hop compounds form Mn^2+^-hop compounds complexes inside the cell (Simpson and Hughes, 1993; Behr and Vogel, 2010), so another possibility is that they form complexes outside the cells if there is sufficient Mn^2+^, and these complexes cannot efficiently penetrate the cytoplasmic membrane. Furthermore, the high concentration of Mn^2+^ is equivalent to MRS, and it is surprising that the physiological response from the FCM experiments with high Mn^2+^ suggest a protective effect against hop after 3 h (**Figure 8A**), but this protection does not correlate with the observed growth, where only the hop tolerant strains grew (**Figure 2**). This indicates a difference between short term and long term effects. In addition, without Mn^2+^ addition, some of the sensitive strains exhibited higher survival than some of the hop tolerant strains after 3 h exposure (**Figure 8A**). This mechanism requires further investigation, but we hypothesize that it may be related to manganese transporters.

The pHi reduction was less pronounced when hop compounds was added together with high amount of Mn^2+^ (**Figure 7B**), which indicates that the protonophoric effect of hop compounds is much decreased, at least in the short term. These results are similar to the FCM experiments (**Figure 8A**), and suggest that extracellular Mn^2+^ provides some degree of short term protection against hop, but the results from growth experiments (**Figure 2**) and pHi measurement (**Figure 7**) demonstrate that manganese protection is only part of the explanation behind hop tolerance, which must include several other factors as suggested previously (Behr et al., 2006; Bergsveinson et al., 2015).

The combination of hop and ethanol resulted in a larger damaged subpopulation after 3 h than each treatment alone (**Figure 9B**). This suggests that ethanol increases the membrane permeability (Barker and Park, 2001), facilitating the immediate intrusion of hop compounds and injury. In contrast, when following the optical density of the investigated strains over time, the hop tolerant strains grew faster and to slightly higher levels when ethanol and hop was present compared to hop alone (**Figure 9A**). This may seem surprising, but supports a previous observation made on solid hop-gradient agar plates, where a higher tolerance toward hop in the presence of ethanol was observed in a number of beer spoilage strains (Haakensen et al., 2009). Changes in membrane fluidity may be involved, as it has been demonstrated that the membrane fluidity decreases when *L. brevis* cells are grown at low pH and even more upon the addition of hop compounds (Behr et al., 2006). Furthermore, Da Silveira et al. (2003) observed that even though the membranes of *Oenococcus oeni* become more fluid and leaky with increasing concentrations of ethanol, intact cells are able to adapt and reduce their membrane fluidity. We therefore assume that, although the combination of ethanol and hop is more lethal, the few individual cells that survive and grow will adapt to the ethanol in the medium, and the consequent reduction in membrane fluidity has the side effect of protecting cells against hop compounds. This phenomenon highlights that in order to predict beer spoilage potential, it is not sufficient to look only at the immediate physiological response, but also the cell division of the small subpopulation of robust cells.

## Conclusion

The results from FM combined with FCM provide important information about the variation in the response of different *L. brevis* strains. Our results show that hop compounds caused membrane damage, intracellular pH decrease, and cell size reduction in *L. brevis* cells. Upon hop exposure at low pH, only a small subpopulation within the hop tolerant strains maintained membrane integrity. It is only these cells that eventually upregulate the pHi and contribute to the subsequent growth. Furthermore, a high amount of Mn^2+^ provides a short term protection as it increased the viability and the pHi of *L. brevis* cells exposed to hop compounds. In the tested strains, a combination of ethanol and hop compounds caused increased immediate damage to the overall population, while the surviving subpopulation exhibited slightly better growth indicating a level of cross-protection. Overall, the combination of FM and FCM enables us to obtain a better understanding of the physiological response of *L. brevis* cells exposed to a beer-like environment. Additionally, FCM has the potential to become a rapid quality control method in breweries, even though this requires further optimization of the equipment.

## Author Contributions

YZ, HS, and SK conceived and designed the study. YZ ran the experiments and the data analysis. All authors contributed to the writing of the manuscript.

## Conflict of Interest Statement

The authors declare that the research was conducted in the absence of any commercial or financial relationships that could be construed as a potential conflict of interest.

## Footnotes

1 http://imagej.nih.gov/ij/
