# Supplementary material for: Heterogeneity between and within Strains of Lactobacillus brevis Exposed to Beer Compounds
Source: Front Microbiol. 2017 Feb 14;8:239. doi: 10.3389/fmicb.2017.00239 (PMC5308056; doi:10.3389/fmicb.2017.00239)
Supplement: Supplementary file 1 [file Image_1.PDF]

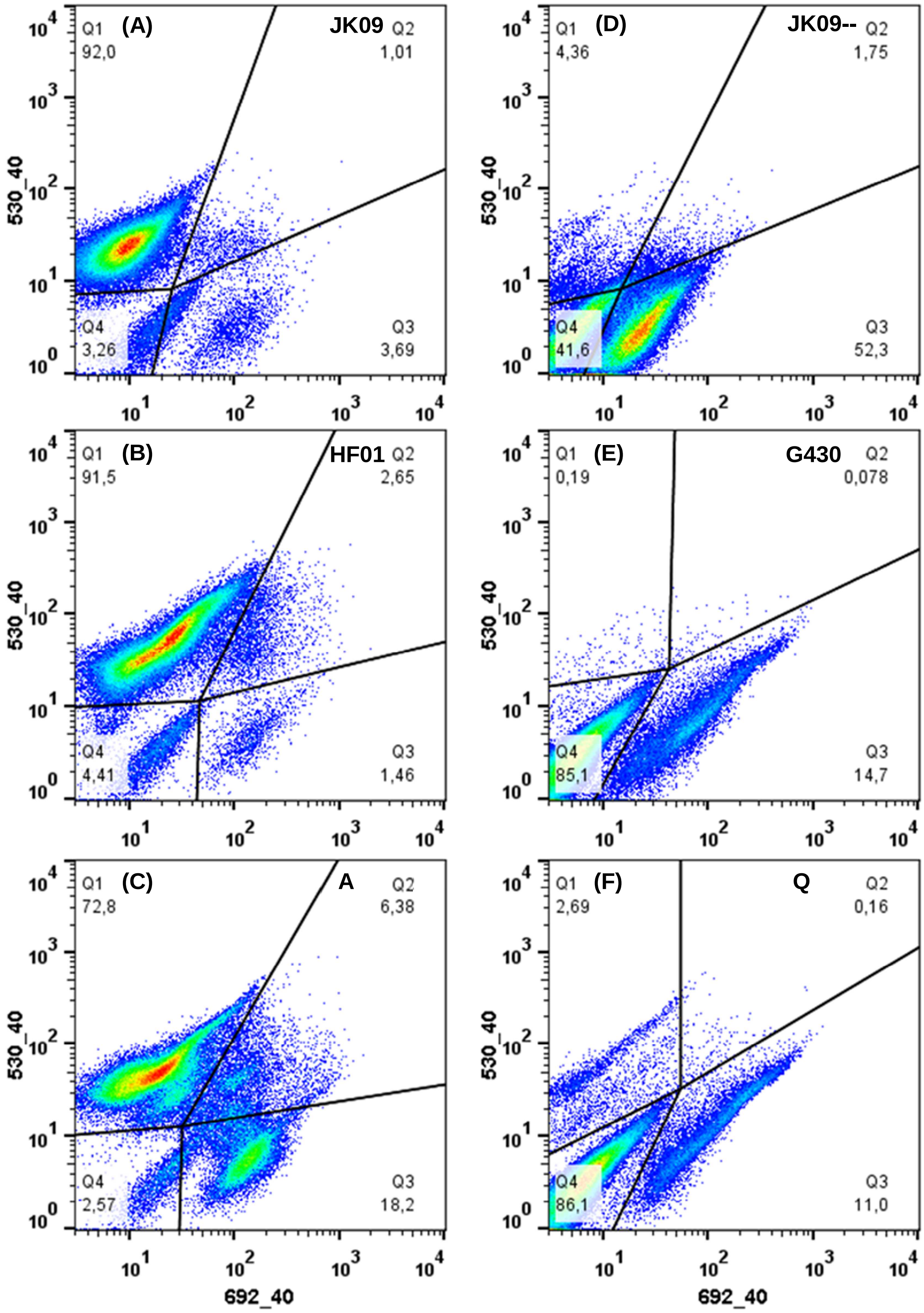

Figure S1 Density plot images of six *L. brevis* strains exposed to hop compounds in $MRS_{4.3}$ after 48h, analyzed by flow cytometry.
